# Change in the Parent-Clinician Relationship Throughout the First Year of Treatment in Pediatric Oncology

**DOI:** 10.1001/jamanetworkopen.2022.30503

**Published:** 2022-09-08

**Authors:** Jennifer W. Mack, Tim Jaung, Hajime Uno, Julienne Brackett

**Affiliations:** 1 Department of Pediatric Oncology, Dana-Farber Cancer Institute, Boston, Massachusetts; 2 Division of Population Sciences’ Center for Outcomes and Policy Research, Dana-Farber Cancer Institute, Boston, Massachusetts; 3 Division of Pediatric Hematology/Oncology, Boston Children’s Hospital, Boston, Massachusetts; 4 Section of Pediatric Hematology/Oncology, Department of Pediatrics, Baylor College of Medicine, Houston, Texas; 5 Department of Pediatrics, Texas Children’s Hospital, Houston, Texas

## Abstract

**Importance:**

Challenges in the therapeutic relationship between clinicians and parents of children with cancer have been shown to emerge immediately after diagnosis, but little is known about whether such relationships improve over time.

**Objective:**

To better understand the potential evolution of parent-clinician relationships over the first year after diagnosis of pediatric cancer.

**Design, Setting, and Participants:**

This survey study was conducted from November 2015 to September 2020 at Dana-Farber Cancer Institute/Boston Children’s Hospital and Texas Children’s Hospital. Participants were oncology clinicians (attending physicians and fellows or nurse practitioners) and parents of children (aged <18 years) with a cancer diagnosis. Surveys were completed at study enrollment (soon after diagnosis [baseline]) and at the 3-month and 12-month follow-up.

**Exposures:**

Children had to have had at least 3 previous clinical visits with a primary oncology clinician.

**Main Outcomes and Measures:**

Survey instruments included the parent and clinician versions of the Relationship Challenges Scale, and factors associated with changes in relationships, including parental, clinician, and health care system attributes, were measured.

**Results:**

Survey participants included 150 parents (118 women [78.7%]; 98 with White race and ethnicity [65.3%]) and 49 clinicians (39 [79.6%] women; 39 with White race and ethnicity [79.6%]). Parents reported on 175 relationships with clinicians, and clinicians reported on 98 relationships with parents at all 3 time points (baseline, 3-month follow-up, and 12-month follow-up). Of the 175 relationships, 33 (18.9%) were considered to be challenging by parents at baseline, 27 (15.4%) were considered to be challenging at the 3-month follow-up, and 32 (18.3%) were considered to be challenging at the 12-month follow-up. Of the 33 challenging relationships at baseline, 20 (60.6%) resolved at the 12-month follow-up, whereas 13 (39.4%) had persistent challenges. However, 19 relationships that were not challenging at baseline had new challenges at the 12-month follow-up, corresponding to 59.4% of all challenges at the 12-month follow-up. No clinician behaviors were associated with improvement. Strategies used frequently (≥50%) by clinicians in their relationships that were associated with improvement included holding family meetings, apologizing, adapting to the parent’s communication style, and devoting extra time and attention.

**Conclusions and Relevance:**

Results of this study showed that some parents experienced challenging relationships with their child’s oncology clinicians beginning at diagnosis and throughout the first year after diagnosis. Although many such relationships improved, others worsened, reflecting the vulnerable and stressful nature of parent-clinician relationships.

## Key Points

**Question:**

How do parent- and clinician-reported challenges in their relationship change over the first year after cancer diagnosis in children?

**Findings:**

In this survey study of 150 parents and 49 oncology clinicians, nearly 20% of parents experienced challenging relationships with clinicians starting at the baseline survey, with a similar proportion of parents reporting the same challenges 1 year later. Although more than 60% of parent-reported relationship challenges had improved by the 12-month survey, a similar proportion of parents experienced new challenges.

**Meaning:**

Findings of this study showed that the therapeutic relationship between clinicians and parents of children with cancer is stressful and vulnerable to difficulties over time, suggesting the need for continued efforts to build supportive relationships throughout treatment.

## Teaser

This survey study assesses changes in the therapeutic relationship between clinicians and parents of children with cancer over the first year of treatment.

## Introduction

A child’s diagnosis of cancer begins a difficult and vulnerable period in a parent’s life,^1,2^ marked by fear and uncertainty about the future^3^ and, often, substantial physical and psychological distress for the child.^4,5,6,7^ As the diagnosis unfolds, parents must entrust the health of their children to oncologists and other clinicians they have just met. This relationship often lasts for years and involves parents and oncology clinicians working together to support the child’s health and well-being.

Not every parent-clinician relationship goes smoothly.^8,9^ In a previous study, we found that nearly one-quarter of parents of children with newly diagnosed cancer experienced challenges in their relationship with clinicians, including threats to trust and mutual understanding.^9^ Similarly, clinicians experienced challenges in more than one-third of their relationships with parents, such as difficulties communicating and delivering the best care.^9^ However, the previous work was conducted shortly after diagnosis, and relationships may potentially heal over time as parents and clinicians learn to work together.

In this study, we sought to better understand the potential evolution of the parent-clinician relationship during the first year after diagnosis of pediatric cancer. Building on previous studies performed at diagnosis,^8,9^ we used parent and clinician surveys at 2 academic cancer centers to identify the prevalence of challenging parent-clinician relationships in pediatric oncology beginning just after diagnosis and continuing for 1 year. Paired surveys were used to compare parent and clinician perspectives, to identify the factors associated with changes in their relationships, and to identify strategies associated with improvement in these relationships.

## Methods

We surveyed parents and oncology clinicians of children with cancer at Dana-Farber Cancer Institute/Boston Children’s Hospital and Texas Children’s Hospital from November 2015 to September 2020. The institutional review boards at both sites approved this survey study and waived the informed consent requirement because this study was deemed to be minimal risk. We followed the American Association for Public Opinion Research (AAPOR) reporting guideline.

Surveys were completed at study enrollment (baseline) and at 3 and 12 months later.^10,11^ Baseline findings have been previously reported.^9^ One parent per child was eligible to participate in the baseline survey. Eligibility included being able to read English or Spanish and having a child younger than 18 years who had had at least 3 visits with a primary oncology clinician and who was within 3 months from receiving a diagnosis at first contact for the baseline survey. Child age and cancer type were identified using medical records.

Primary oncology clinicians were asked for permission to contact the parents, and the clinicians for 12 parents declined. Eligible parents were contacted in person or by mail and were given a letter inviting them to participate along with the parent version of the baseline survey questionnaire (Relationship Challenges Scale–Parent) and a postage-paid, opt-out postcard. The survey was framed as a study about communication between parents and clinicians with a goal of understanding what helps communication go well and what makes it challenging. Both the English and Spanish versions of the survey questionnaires were available in paper and electronic formats. Nonrespondents were contacted 2 times in person or by mail or telephone. Respondents received a $10 gift card.

In the parent version of the baseline survey, parents were asked to name up to 2 primary oncology clinicians involved in their child’s care according to the care systems at the sites wherein an attending physician and a fellow or nurse practitioner often work together to fulfill this role. Parents were asked to answer questions about each clinician relationship separately.

After the parents completed their survey, the clinician version of the survey (Relationship Challenges Scale–Clinician) was provided to the clinicians named by the parents. This version asked about the clinicians' relationship with parents. Respondents received a $5 gift card.

Parents were eligible to participate in the longitudinal follow-up surveys at 3 and 12 months if they and the clinicians they named completed their paired surveys at the previous time point (baseline). In both surveys at the 3- and 12-month follow-up, parents were asked about their relationship with the clinicians. Respondents received a $10 gift card for each survey. Clinicians were then offered to complete the survey at the 3- and 12-month follow-up. Respondents received a $5 gift card for each survey.

### Parent Surveys

The Relationship Challenges Scale–Parent version was developed for this study.^8,12^ Cognitive interviews with 10 parents of children with cancer confirmed the conceptual, face, and content validity of the questionnaire. It included 11 questions on listening, sensitivity, mutual respect, and trust (Cronbach α = .87), and responses were specified on a 4-point Likert scale.^9^

The baseline questionnaire asked parents to identify their sex, race and ethnicity, educational level, and primary language. Race and ethnicity were collected based on previous data identifying suboptimal communication experiences among parents of racial or ethnic minority status. The Hospital Anxiety and Depression Scale was also used to self-evaluate depression and anxiety at each time point (baseline, 3-month follow-up, and 12-month follow-up), with scores of 8 or higher on each subscale considered to be suggestive of a condition.^13^

Parents were asked how well other physicians and nurses involved in their child’s care worked together (interdisciplinary teamwork), how often they were told different things by different people (mixed messages), how often different physicians and nurses were aware of the child’s medical history (communication across transitions), and how often different physicians and nurses were aware of the child’s special needs (patient-centeredness across transitions).^14^ Response options were always, often, sometimes, rarely, or never.

### Clinician Surveys

The Relationship Challenges Scale–Clinician version was developed for this study.^8,15,16,17^ Cognitive interviews with 10 pediatric oncology clinicians confirmed the conceptual, face, and content validity of the questionnaire. It included 6 questions on challenges related to communicating and delivering best care (Cronbach α = .82). A 6-point Likert scale, ranging from not at all to a great deal, was used to specify responses. Clinicians completed the questionnaire for each parent who had named them in the parent survey.

The baseline survey asked clinicians to identify their role (attending physician, fellow, or nurse practitioner), sex, race and ethnicity, and years in practice. In addition, clinicians were asked whether they had used strategies to work with the parent, including holding family meetings, apologizing, setting limits or boundaries, adapting to the parent’s communication style, devoting extra time and attention, and seeking help from other clinicians.^9^

### Statistical Analysis

For the Relationship Challenges Scale–Parent version, we considered relationships to be challenging if a parent responded to any single question in the 2 lowest of 4 possible categories, similar to existing patient experience measures.^9,14,18,19,20^ For the clinician version of the survey, we considered challenges to be present if a clinician responded to any question in the 3 lowest of 6 possible response categories.^9^

Proportions of parents and clinicians reporting challenging relationships at each time point were calculated, and marginal asymmetry between parent and clinician perspectives was ascertained using the McNemar test. Mixed-effects logistic regression models were used to examine changes over time in parent and clinician reports of challenging relationships after adjustment for associated parent, clinician, and health care systems factors.

Multinomial mixed-effects logistic regression was used to identify factors associated with patterns of change in challenges over time, with relationships classified into 3 groups: (1) no challenges at baseline; (2) challenges at baseline that resolved with time; and (3) challenges at baseline that persisted. Multinomial models of parent-identified challenges examined the factors associated with the resolution of challenges between baseline and the 12-month follow-up as well as the factors associated with persistent challenges between baseline and the 12-month follow-up compared with parents who did not identify challenges at baseline.

Models of clinician-identified challenges used an analogous approach. Bivariable and multivariable mixed-effects models were created, accounting for 1 to 2 relationships per parent and multiple possible relationships per clinician. Factors associated with parent-identified challenges were evaluated for 175 relationships, with parent reports at all 3 time points. Factors associated with clinician-identified challenges were evaluated in 98 relationships, with clinician reports at all 3 time points.

Covariates were dichotomized. Because of the small numbers of parents who self-identified as having Asian, Black, Hispanic, or other race and ethnicity, race and ethnicity were categorized for analysis as White non-Hispanic (the largest group) vs racial and ethnic minority group. Candidate variables were based on previous findings and reports at baseline, including parent sociodemographic factors and mental health, clinician factors, and health care systems factors, such as care across transitions.^9^ A backward variable selection procedure was used to construct the multivariable model, with a significance level of *P* = .05 for removal from the model. Mixed-effects multinomial logistic regression models were also used to examine the association between strategies used by clinicians to work with parents and patterns of change in challenges over time.

A 2-sided *P* < .05 was used for statistical significance. Statistical analyses were conducted using R, version 4.0.2 (R Foundation for Statistical Computing).

## Results

Surveys were completed by 150 parents (118 women [78.7%] and 32 men [21.3%]; 98 with White race and ethnicity [65.3%]) and 49 clinicians (39 [79.6%] women and 10 men [20.4%]; 39 with White race and ethnicity [79.6%]) (Table 1). Nearly half of the clinicians were attending physicians (24 [49.0%]), whereas 17 were fellows (34.7%) and 8 were nurse practitioners (16.3%).

**Table 1.** Parent, Child, and Clinician Characteristics

Characteristic	Parents with complete longitudinal surveys	Parents with paired complete longitudinal clinician surveys
**Parent characteristics**		
No.	150	91
Sex		
Female	118 (78.7)	70 (76.9)
Male	32 (21.3)	21 (23.1)
Race and ethnicity^a^		
Asian	8 (5.3)	7 (7.7)
Black	6 (4.0)	3 (3.3)
Hispanic	27 (18.0)	19 (20.9)
White	98 (65.3)	54 (59.3)
Other^b^	6 (4.0)	5 (5.5)
Missing data	5 (3.3)	3 (3.3)
Age group, y		
<30	20 (13.3)	13 (14.3)
30-39	70 (46.7)	38 (41.8)
40-49	42 (28.0)	29 (31.9)
≥50	15 (10.0)	8 (8.8)
Missing data	3 (2.0)	3 (3.3)
Educational level		
≤High school diploma	27 (18.0)	16 (17.6)
>High school diploma	121 (80.7)	73 (80.2)
Missing data	2 (1.3)	2 (2.2)
Marital status		
Married or living as married	116 (77.3)	69 (75.8)
Not married or not living as married	33 (22.0)	21 (23.1)
Missing data	1 (0.7)	1 (1.1)
Primary language spoken at home		
English	119 (79.3)	69 (75.8)
Spanish	6 (4.0)	3 (3.3)
Other^b^	2 (1.3)	2 (2.2)
Bilingual		
English and Spanish	3 (2.0)	2 (2.2)
English and other	5 (3.3)	4 (4.4)
Missing data	15 (10.0)	11 (12.1)
**Child characteristics**		
No.	150	91
Age group, y		
<2	15 (10.0)	9 (9.9)
2-4	40 (26.7)	27 (29.7)
5-7	30 (20.0)	16 (17.6)
8-10	10 (6.7)	6 (6.6)
11-17	55 (36.7)	33 (36.3)
Cancer type		
Hematologic	105 (70.0)	62 (68.1)
Brain or solid tumor	41 (27.3)	29 (31.9)
**Clinician characteristics**		
No.	49	NA
Sex		
Female	39 (79.6)	NA
Male	10 (20.4)	NA
Race and ethnicity^a^		
Asian	7 (14.3)	NA
Black	1 (2.0)	NA
Hispanic	2 (4.1)	NA
White	39 (79.6)	NA
Clinician role		
Attending physician	24 (49.0)	NA
Fellow	17 (34.7)	NA
Nurse practitioner	8 (16.3)	NA
Hours per week in clinical care		
<10	6 (12.2)	NA
10-19	11 (22.4)	NA
≥20	32 (65.3)	NA
Years since graduation from medical or nursing school		
<10	22 (44.9)	NA
10-19	16 (32.7)	NA
≥20	9 (18.4)	NA
Missing data	2 (4.1)	NA

Abbreviation: NA, not applicable.

^a^ Race and ethnicity were self-identified by participants.

^b^ Given the small number of individuals in the other categories, “other” is not specified to protect the identity of participants.

The baseline survey was completed by 400 parents (77.7% of 515 eligible), with clinician surveys available for 273 parents who were then eligible for the longitudinal follow-up surveys. Of the 273 parents, 263 (96.3%) completed the 3-month follow-up survey, with paired clinician surveys available for 197 parents. A total of 150 parents (55.0% of 273 eligible for longitudinal follow-up surveys and 76.1% of 197 with paired 3-month follow-up surveys) completed the surveys across all 3 time points, providing reports on 175 relationships. A total of 91 parents had paired clinician surveys at all 3 time points, corresponding to 98 relationships and 49 different clinicians. Parents with complete longitudinal data were not statistically different from those with baseline data with respect to parent sex, race and ethnicity, and educational level (Table 1).

### Change in Parent-Identified Challenging Relationships Over Time

Of the 175 relationships with completed parental surveys for all 3 time points, 33 (18.9%) were identified as challenging by parents at baseline as were 27 (15.4%) at the 3-month follow-up and 32 (18.3%) at the 12-month follow-up. In a mixed-effects logistic regression model, the overall proportion of relationships considered to be challenging by parents had no significant linear time pattern (odds ratio [OR], 1.71; 95% CI, 0.89-3.29; *P* = .11) after adjustment for parental depression (OR, 2.72; 95% CI, 1.37-5.40; *P* = .004) and parental report of mixed messages (OR, 3.54; 95% CI, 1.77-7.10; *P* < .001). However, many parent-clinician relationships did change over time. Of the 33 relationships reported as challenging at baseline, 20 (60.6%) were no longer challenging at the 12-month follow-up, whereas 13 (39.4%) had persistent challenges. In addition, 19 relationships that were not challenging at baseline had new challenges at the 12-month follow-up, corresponding to 59.4% of all challenges at the 12-month follow-up.

In the bivariable multinomial mixed-effects logistic regression, parents with a high school diploma or lower educational level (OR, 2.82; 95% CI, 1.01-7.90; *P* = .049) and those with anxiety (OR, 2.84; 95% CI, 1.07-7.57; *P* = .04) were more likely to experience challenges at baseline that resolved with time compared with parents who reported no challenges at baseline (Table 2). Parents who reported inconsistent communication across transitions were more likely to experience persistent challenges (OR, 4.50; 95% CI, 1.02-19.93; *P* = .048). Parents who identified as being from a racial and ethnic minority group also had greater absolute odds of persistent challenges over time, although the OR did not meet statistical significance.

**Table 2.** Factors Associated With Relationship Challenges Identified by Parents and Clinicians

Variable	Parent-identified challenges (n = 175 relationships)		Clinician-defined challenges (n = 98 relationships)	
	OR (95% CI)	*P* value	OR (95% CI)	*P* value
**Parental factors**				
Male parent				
No challenges at baseline	1 [Reference]		1 [Reference]	
Challenges at baseline that resolved with time	1.47 (0.52-4.14)	.46	1.24 (0.41-3.80)	.71
Challenges at baseline that persisted	1.03 (0.27-3.99)	.97	0.58 (0.11-2.91)	.51
Parent from a racial or ethnic minority group				
No challenges at baseline	1 [Reference]		1 [Reference]	
Challenges at baseline that resolved with time	1.42 (0.52-3.92)	.50	1.52 (0.50-4.69)	.46
Challenges at baseline that persisted	3.13 (0.94-10.43)	.06	0.75 (0.14-4.03)	.74
Parent with ≤high school diploma educational level				
No challenges at baseline	1 [Reference]		1 [Reference]	
Challenges at baseline that resolved with time	2.82 (1.01-7.90)	.049	1.92 (0.57-6.49)	.30
Challenges at baseline that persisted	3.03 (0.90-10.15)	.07	1.84 (0.41-8.16)	.42
Parent with non-English primary language				
No challenges at baseline	1 [Reference]		1 [Reference]	
Challenges at baseline that resolved with time	0.46 (0.06-3.74)	.47	2.30 (0.74-7.14)	.15
Challenges at baseline that persisted	1.55 (0.30-7.96)	.60	4.57 (1.24-16.81)	.02
Parental anxiety				
No challenges at baseline	1 [Reference]		1 [Reference]	
Challenges at baseline that resolved with time	2.84 (1.07-7.57)	.04	1.65 (0.55-4.97)	.37
Challenges at baseline that persisted	2.07 (0.63-6.78)	.23	0.60 (0.14-2.47)	.48
Parental depression				
No challenges at baseline	1 [Reference]		1 [Reference]	
Challenges at baseline that resolved with time	1.98 (0.74-5.31)	.18	1.22 (0.32-4.63)	.78
Challenges at baseline that persisted	1.95 (0.57-6.61)	.29	1.36 (0.32-5.89)	.68
**Clinician factors**				
Fellow or nurse practitioner				
No challenges at baseline	1 [Reference]		1 [Reference]	
Challenges at baseline that resolved with time	0.93 (0.34-2.50)	.88	2.79 (1.07-7.27)	.04
Challenges at baseline that persisted	0.99 (0.31-3.19)	.99	1.14 (0.34-3.87)	.83
Clinician male sex				
No challenges at baseline	1 [Reference]		1 [Reference]	
Challenges at baseline that resolved with time	1.04 (0.32-3.37)	.95	1.62 (0.44-5.92)	.47
Challenges at baseline that persisted	1.17 (0.30-4.55)	.82	1.92 (0.50-7.37)	.34
Clinician with 10-19 (vs <10) years in practice				
No challenges at baseline	1 [Reference]		1 [Reference]	
Challenges at baseline that resolved with time	1.78 (0.54-5.83)	.34	1.16 (0.34-3.92)	.82
Challenges at baseline that persisted	1.43 (0.38-5.31)	.59	3.50 (0.77-15.88)	.10
Clinician with ≥20 (vs <10) years in practice				
No challenges at baseline	1 [Reference]		1 [Reference]	
Challenges at baseline that resolved with time	1.67 (0.44-6.32)	.45	0.67 (0.18-2.44)	.54
Challenges at baseline that persisted	1.07 (0.23-5.11)	.93	1.40 (0.28-7.06)	.68
**Health care system factors**				
Poor interdisciplinary teamwork				
No challenges at baseline	1 [Reference]		NA^a^	
Challenges at baseline that resolved with time	3.72 (0.32-43.16)	.29	NA	NA
Challenges at baseline that persisted	6.09 (0.51-73.19)	.16	NA	NA
Mixed messages				
No challenges at baseline	1 [Reference]		NA^a^	
Challenges at baseline that resolved with time	2.17 (0.65-7.19)	.21	NA	NA
Challenges at baseline that persisted	0.98 (0.19-4.95)	.98	NA	NA
Inconsistent communication across transitions				
No challenges at baseline	1 [Reference]		1 [Reference]	
Challenges at baseline that resolved with time	3.36 (0.80-14.11)	.10	1.81 (0.50-6.58)	.37
Challenges at baseline that persisted	4.50 (1.02-19.93)	.048	1.25 (0.23-6.84)	.8
Poor patient-centeredness across transitions				
No challenges at baseline	1 [Reference]		1 [Reference]	
Challenges at baseline that resolved with time	1.79 (0.36-9.03)	.48	2.54 (0.45-14.47)	.29
Challenges that persisted	2.87 (0.54-15.30)	.22	3.21 (0.48-21.54)	.23

Abbreviations: NA, not available; OR, odds ratio.

^a^ Model could not be run because of 0 count for an included outcome.

In a multivariable model, parents with a high school diploma or lower educational level were more likely to experience both challenges at baseline that resolved with time (adjusted OR [aOR], 4.25; 95% CI, 1.26-14.28; *P* = .02) and persistent challenges (aOR, 4.29; 95% CI, 1.12-16.36; *P* = .03). A similar pattern was seen for parents who experienced inconsistent communication across transitions (resolved challenges: aOR, 5.04 [95% CI, 1.09-23.34], *P* = .04; persistent challenges: aOR, 6.23 [95% CI, 1.25-31.00], *P* = .03).

### Change in Clinician-Identified Challenging Relationships Over Time

**Figure 1.**
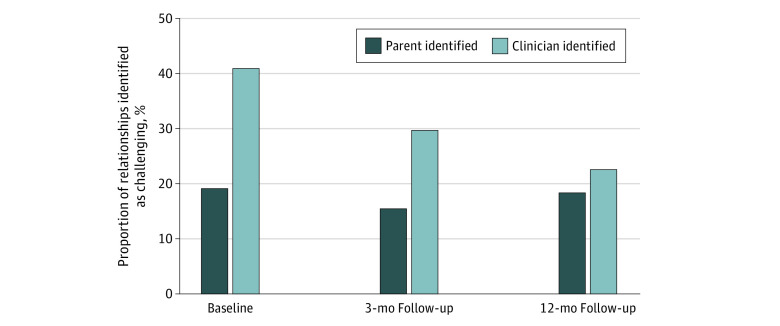
Proportion of Relationships Identified as Challenging by Parents and Clinicians at Each Time Point Parents reported 175 relationships, and clinicians reported 98 relationships.

Paired patient-clinician surveys were completed for 98 relationships. The proportion of relationships considered to be challenging by clinicians ranged from 40.8% (40) at baseline to 29.6% (29) at the 3-month follow-up to 22.4% (22) at the 12-month follow-up (Figure 1). In a mixed-effects logistic regression model, the proportion considered to be challenging by clinicians decreased with time (OR, 0.64; 95% CI, 0.46-0.89; *P* < .01) after adjustment for parental report of mixed messages (OR, 2.67; 95% CI, 1.42-5.02; *P* < .01). When compared at each time point, parental and clinician perspectives on challenging relationships were often not aligned. Relationships were identified as challenging by both parents and clinicians for 11 of 98 (11.2%) relationships at baseline, 6 relationships (6.1%) at the 3-month follow-up, and 7 relationships (7.1%) at the 12-month follow-up.

When perspectives were not aligned, clinicians tended to report more challenges at baseline and at the 3-month follow-up. At baseline, 29% of clinician challenges were not reported by parents compared with 11% of parent challenges that were not reported by clinicians. At the 3-month follow-up, 23% of clinician challenges were not reported by parents compared with 10% of parent challenges that were not reported by clinicians. By the 12-month follow-up, reports of challenging relationships were evenly distributed between parents and clinicians. That is, 15% of clinician challenges were not reported by parents compared with 12% of parent challenges that were not reported by clinicians.

In bivariable analyses from the perspective of clinicians, persistent challenges were more likely to occur when parents used a primary language other than English (OR, 4.57; 95% CI, 1.24-16.81; *P* = .02). Fellows and nurse practitioners were more likely to have early challenges that resolved with time (OR, 2.79; 95% CI, 1.07-7.27; *P* = .04) compared with attending physicians, a finding that remained the sole factor after a multivariable model was created (aOR, 2.79; 95% CI, 1.07-7.27; *P* = .04).

### Clinician Strategies Used to Manage Relationship Challenges

**Figure 2.**
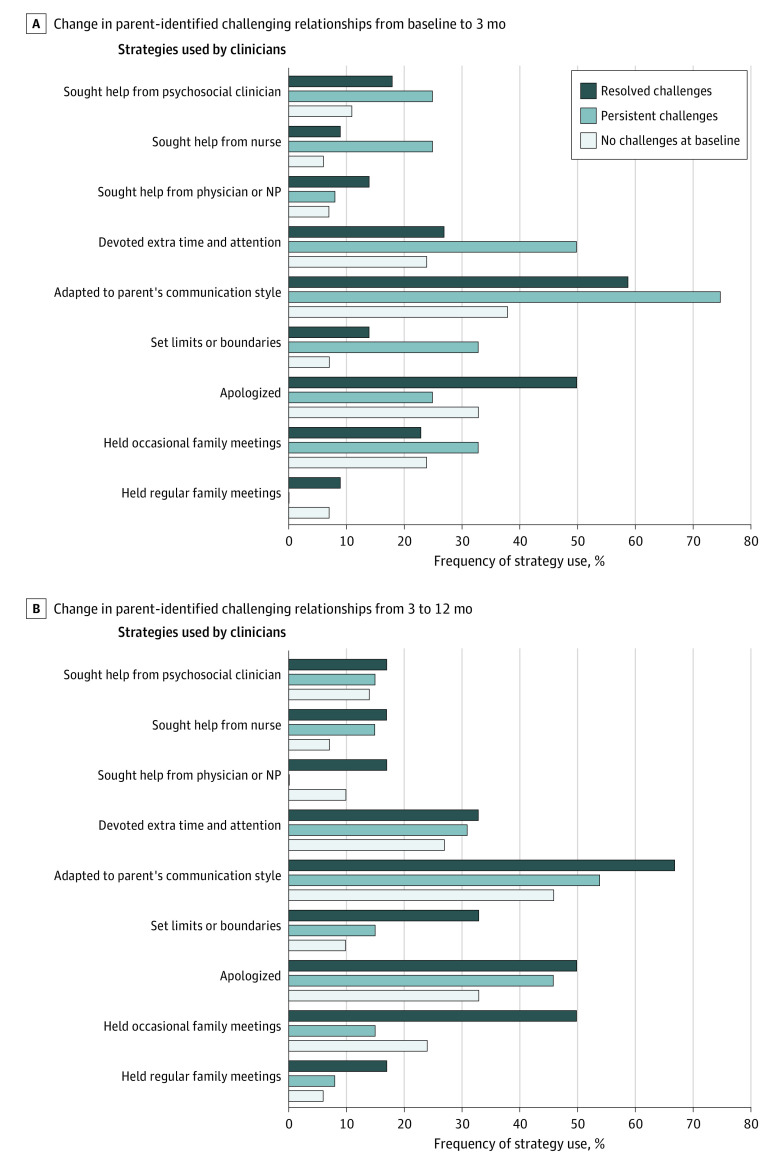
Association Between Strategies Used by Clinicians and Improvement in Parent-Identified Challenging Relationships Over Time NP indicates nurse practitioner.

We also examined strategies used by clinicians to manage relationships. No strategies were consistently associated with improved parent-identified challenging relationships (Figure 2). However, some strategies were used frequently (≥50%) in relationships that demonstrated improvement, including holding occasional family meetings (changed from baseline to 3-month follow-up), apologizing (changed from 3- to 12-month follow-up), adapting to the parent’s communication style (changed from 3- to 12-month follow-up), and devoting extra time and attention (changed from 3- to 12-month follow-up).

**Figure 3.**
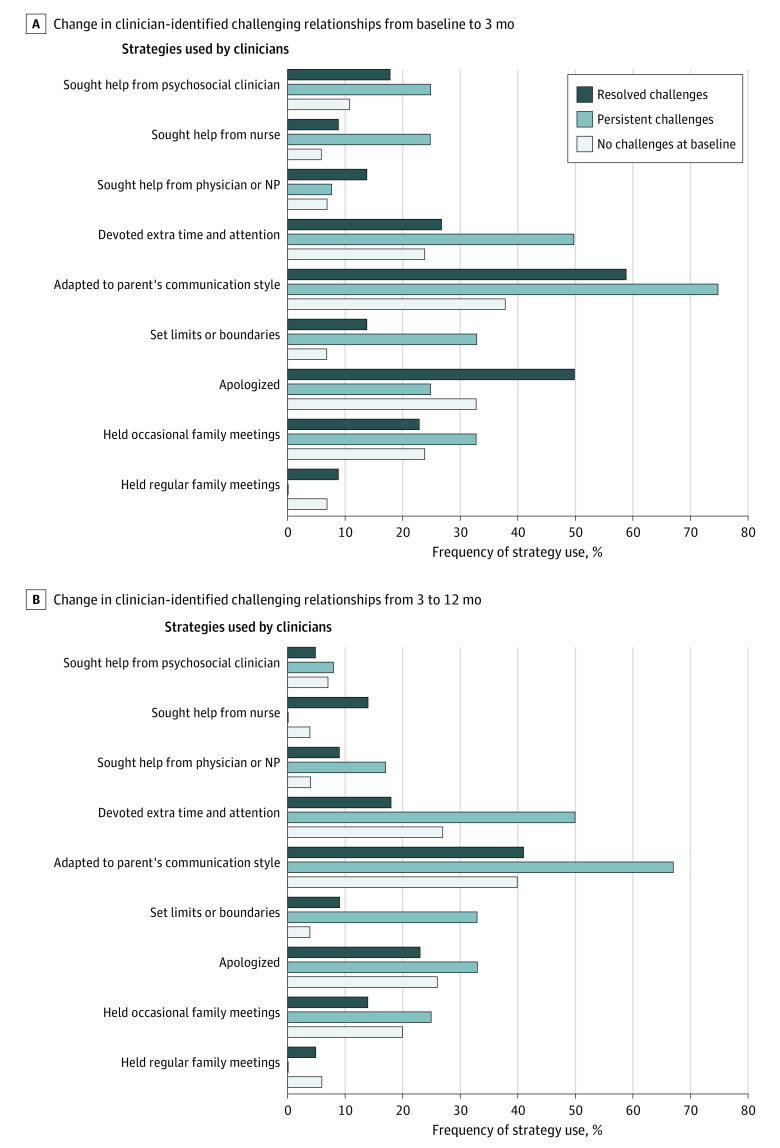
Association Between Strategies Used by Clinicians and Improvement in Clinician-Identified Challenging Relationships Over Time NP indicates nurse practitioner.

When clinicians considered relationships to be challenging, they were more likely to report setting limits at the 3-month follow-up (persistent challenges: 33%; no challenges at baseline: 7%; resolved challenges: 14%; *P* = .04) and 12-month follow-up (persistent challenges: 33%; no challenges at baseline: 4%; resolved challenges: 9%; *P* = .01), with higher proportions in relationships that remained challenging (Figure 3). Clinicians were also more likely to report adapting to the parent’s communication style at the 3-month follow-up, with higher proportions in relationships that remained challenging (persistent challenges: 75%; no challenges at baseline: 38%; resolved challenges: 59%; *P* = .04) (Figure 3).

## Discussion

The therapeutic relationship is a fundamental aspect of patient-centered care.^21,22,23^ Our previous work found that not all parents of children with cancer experienced supportive relationships with their children’s oncology clinicians, with nearly one-quarter of these parents identifying substantial challenges soon after diagnosis.^9^ Such relationships can evolve; however, in the best case, a rough start can become smoother with time.

In this survey study, the proportion of relationships that parents identified as challenging remained stable over time. Although some relationships improved, others developed new challenges, with 18.9% of relationships experiencing challenges at any time over the first year after diagnosis. Lower educational levels of parents and communication challenges across health care transitions were associated with early challenges that resolved with time. However, these same factors were also associated with early challenges that persisted; in other words, these factors put parents at risk for early challenges, some of which improved and some of which did not.

Although we examined clinician behaviors, such as apologizing or holding family meetings, none of these strategies were associated with improvement in parent-reported challenges. Given the small sample of parents with baseline challenges, the study had limited power to detect associations. Nonetheless, some behaviors were present in at least one-half of the relationships that improved, including apologizing, holding occasional family meetings, devoting extra time and attention, and adapting to the parent’s communication style. Although the study design did not allow for cause-and-effect analysis and the study had limited statistical power, these caring communication behaviors could be further evaluated as potential pathways to improve relationships where there are struggles.

Furthermore, the challenges defined by clinicians were prevalent, affecting 40.8% of clinician relationships at baseline. Fellows and nurse practitioners, who often represent the front line of clinical care, were particularly affected. Clinicians appeared to take self-protective stances by setting limits and boundaries, which might be a strategy when parents engage in strong advocacy, and this choice was most prevalent in relationships with persistent challenges. Given the finding of high burnout among clinicians in challenging relationships,^9^ the high prevalence soon after diagnosis underscored the stress experienced by clinicians in pediatric oncology. Yet by the 12-month follow-up, the number of relationships with challenges had decreased by nearly half. This decrease suggests that clinicians who are able to keep moving forward in relationships often, but not always, find a way to work through challenges. In addition, some challenges may naturally diminish with time after the stress of diagnosis. Future work to identify strategies to recognize and navigate challenges may enhance the resilience of and reduce burnout among clinicians.

### Limitations

This study has several limitations. We lacked longitudinal data for many parents who participated at baseline. As a result, we had limited power to assess the strategies to improve relationships, and we do not know whether the experiences identified by the parents in this study are representative of the experiences of the larger parent population. We experienced attrition over time, and those with relationship challenges may have been less likely to respond, especially if they changed institutions or clinicians as a result. The study also lacked statistical power to evaluate the factors associated with the development of new challenges in relationships after baseline; this topic deserves attention in future work. Because of limited racial and ethnic diversity in this sample and the small sample size, we combined the racial and ethnic minority groups for analysis, thereby losing the ability to detect important differences between populations. Our previous work, which focused on baseline findings, demonstrated racial and ethnic differences in relationship quality.^9^ Although there was some suggestion of increased early challenges among parents who reported being from a racial or ethnic minority group, the finding did not meet statistical significance, perhaps partly because of the use of a consolidated measure and the sample size. Moreover, male parents were underrepresented. Future work is needed to explore the experiences of historically marginalized populations and to address and potentially meet the care needs of all patients and family members in pediatric oncology.

## Conclusions

Clinicians can take away 4 main points from the findings of this study. First, relationships are fluid, and the relationship challenges that clinicians experience with parents can often improve with time. Second, although some of these relationships may improve, others may worsen or experience new challenges, underscoring the vulnerable, stressful, disruptive nature of parent-clinician relationships throughout the first year of a pediatric cancer diagnosis and likely beyond. Third, parents with lower educational levels are at a heightened risk for challenges, suggesting that parental needs require special attention. Fourth, caring, compassionate behaviors, such as apologizing when things do not go well, holding family meetings, devoting time and attention, and adapting to the parent’s communication style, are important strategies for every clinician’s ongoing practice. Working together with patients and families to build supportive relationships is a worthy endeavor for all clinicians who care for children with serious illness.

## References

[1] Kearney JA, Salley CG, Muriel AC. Standards of psychosocial care for parents of children with cancer. Pediatr Blood Cancer. 2015;62(suppl 5):S632-S683. doi:10.1002/pbc.25761 26700921 PMC5066591

[2] Kazak AE, Stuber ML, Barakat LP, Meeske K, Guthrie D, Meadows AT. Predicting posttraumatic stress symptoms in mothers and fathers of survivors of childhood cancers. J Am Acad Child Adolesc Psychiatry. 1998;37(8):823-831. doi:10.1097/00004583-199808000-00012 9695444

[3] Greenzang KA, Cronin AM, Kang TI, Mack JW. Parental distress and desire for information regarding long-term implications of pediatric cancer treatment. Cancer. 2018;124(23):4529-4537. doi:10.1002/cncr.31772 30276800 PMC6289673

[4] Katz ER, Jay S. Psychological aspects of cancer in children, adolescents, and their families. Clin Psychol Rev. 1984;4(5):525-542. doi:10.1016/0272-7358(84)90042-4

[5] Hooke MC, Linder LA. Symptoms in children receiving treatment for cancer-part I: fatigue, sleep disturbance, and nausea/vomiting. J Pediatr Oncol Nurs. 2019;36(4):244-261. doi:10.1177/1043454219849576 31307321 PMC7197223

[6] Linder LA, Hooke MC. Symptoms in children receiving treatment for cancer-part II: pain, sadness, and symptom clusters. J Pediatr Oncol Nurs. 2019;36(4):262-279. doi:10.1177/1043454219849578 31307323 PMC7197222

[7] Wolfe J, Grier HE, Klar N, . Symptoms and suffering at the end of life in children with cancer. N Engl J Med. 2000;342(5):326-333. doi:10.1056/NEJM200002033420506 10655532

[8] Mack JW, Ilowite M, Taddei S. Difficult relationships between parents and physicians of children with cancer: a qualitative study of parent and physician perspectives. Cancer. 2017;123(4):675-681. doi:10.1002/cncr.30395 27727442

[9] Mack JW, Jaung T, Uno H, Brackett J. Parent and clinician perspectives on challenging parent-clinician relationships in pediatric oncology. JAMA Netw Open. 2021;4(11):e2132138. doi:10.1001/jamanetworkopen.2021.32138 34787658 PMC8600390

[10] Feraco AM, Brand SR, Gagne J, Sullivan A, Block SD, Wolfe J. Development of the “Day 100 Talk”: addressing existing communication gaps during the early cancer treatment period in childhood cancer. Pediatr Blood Cancer. 2018;65(6):e26972. doi:10.1002/pbc.26972 29384265 PMC5911188

[11] Sisk BA, Kang TI, Mack JW. Prognostic disclosures over time: parental preferences and physician practices. Cancer. 2017;123(20):4031-4038. doi:10.1002/cncr.30716 28369836

[12] Mack JW, Block SD, Nilsson M, . Measuring therapeutic alliance between oncologists and patients with advanced cancer: the Human Connection Scale. Cancer. 2009;115(14):3302-3311. doi:10.1002/cncr.24360 19484795 PMC2771331

[13] Spinhoven P, Ormel J, Sloekers PP, Kempen GI, Speckens AE, Van Hemert AM. A validation study of the Hospital Anxiety and Depression Scale (HADS) in different groups of Dutch subjects. Psychol Med. 1997;27(2):363-370. doi:10.1017/S0033291796004382 9089829

[14] Cleary PD, Edgman-Levitan S, Roberts M, . Patients evaluate their hospital care: a national survey. Health Aff (Millwood). 1991;10(4):254-267. doi:10.1377/hlthaff.10.4.254 1778560

[15] Hahn SR. Physical symptoms and physician-experienced difficulty in the physician-patient relationship. Ann Intern Med. 2001;134(9, pt 2):897-904. doi:10.7326/0003-4819-134-9_Part_2-200105011-00014 11346326

[16] Hahn SR, Kroenke K, Spitzer RL, . The difficult patient: prevalence, psychopathology, and functional impairment. J Gen Intern Med. 1996;11(1):1-8. doi:10.1007/BF02603477 8691281

[17] Hahn SR, Thompson KS, Wills TA, Stern V, Budner NS. The difficult doctor-patient relationship: somatization, personality and psychopathology. J Clin Epidemiol. 1994;47(6):647-657. doi:10.1016/0895-4356(94)90212-7 7722577

[18] Dyer N, Sorra JS, Smith SA, Cleary PD, Hays RD. Psychometric properties of the Consumer Assessment of Healthcare Providers and Systems (CAHPS®) clinician and group adult visit survey. Med Care. 2012;50(suppl):S28-S34. doi:10.1097/MLR.0b013e31826cbc0d 23064274 PMC3480671

[19] Homer CJ, Fowler FJ, Gallagher PM, . The Consumer Assessment of Health Plan Study (CAHPS) survey of children’s health care. Jt Comm J Qual Improv. 1999;25(7):369-377. doi:10.1016/S1070-3241(16)30452-7 10412084

[20] Robert Wood Johnson Foundation. Developing a public report for the CAHPS clinician & group survey: a decision guide. October 2013. Accessed August 3, 2022. https://www.rwjf.org/en/library/research/2013/09/developing-a-public-report-for-the-cahps-clinician---group-surve.html

[21] Hewitt M, Simone J, eds. Ensuring Quality Cancer Care. National Academies Press; 1999. 25101447

[22] Cleary PD, McNeil BJ. Patient satisfaction as an indicator of quality care. Inquiry. 1988;25(1):25-36. 2966123

[23] Epstein R, Street R Jr. Patient-Centered Communication in Cancer Care: Promoting Healing and Reducing Suffering. National Cancer Institute: 2007.

